# Social Network Analysis and Mining to Monitor and Identify Problems with Large-Scale Information and Communication Technology Interventions

**DOI:** 10.1371/journal.pone.0146220

**Published:** 2016-01-04

**Authors:** Aleksandra do Socorro da Silva, Silvana Rossy de Brito, Nandamudi Lankalapalli Vijaykumar, Cláudio Alex Jorge da Rocha, Maurílio de Abreu Monteiro, João Crisóstomo Weyl Albuquerque Costa, Carlos Renato Lisboa Francês

**Affiliations:** 1 Cyberspace Institute, Federal Rural University of Amazon, Pará, Brazil; 2 Special Technologies Center, National Institute for Space Research, São Paulo, Brazil; 3 Informatics, Federal Institute of Education, Science and Technology of Pará, Pará, Brazil; 4 Amazon Higher Studies Nucleus, Federal University of Pará, Pará, Brazil; 5 Institute of Technology, Federal University of Pará, Pará, Brazil; University of Cape Town, SOUTH AFRICA

## Abstract

The published literature reveals several arguments concerning the strategic importance of information and communication technology (ICT) interventions for developing countries where the digital divide is a challenge. Large-scale ICT interventions can be an option for countries whose regions, both urban and rural, present a high number of digitally excluded people. Our goal was to monitor and identify problems in interventions aimed at certification for a large number of participants in different geographical regions. Our case study is the training at the Telecentros.BR, a program created in Brazil to install telecenters and certify individuals to use ICT resources. We propose an approach that applies social network analysis and mining techniques to data collected from Telecentros.BR dataset and from the socioeconomics and telecommunications infrastructure indicators of the participants’ municipalities. We found that (i) the analysis of interactions in different time periods reflects the objectives of each phase of training, highlighting the increased density in the phase in which participants develop and disseminate their projects; (ii) analysis according to the roles of participants (i.e., tutors or community members) reveals that the interactions were influenced by the center (or region) to which the participant belongs (that is, a community contained mainly members of the same region and always with the presence of tutors, contradicting expectations of the training project, which aimed for intense collaboration of the participants, regardless of the geographic region); (iii) the social network of participants influences the success of the training: that is, given evidence that the *degree* of the community member is in the highest range, the probability of this individual concluding the training is 0.689; (iv) the North region presented the lowest probability of participant certification, whereas the Northeast, which served municipalities with similar characteristics, presented high probability of certification, associated with the highest *degree* in social networking platform.

## Introduction

Information and communication technologies (ICTs) influence how individuals, companies, and society perform their functions. There are several arguments in favor of the use of ICTs, including its strategic importance for increasing the competitiveness of business organizations [1,2]; e-commerce, e-business, and new business models [3,4]; improvement of social services and governance [5,6]; changes in public policies [7]; modernization of public management [8,9]; improvement in health care [10,11] and education systems [12]; and expansion of democratic participation [13,14].

Developing countries have a growing interest in implementing ICT intervention in urban and rural areas, either to increase the development of the country or to decrease internal inequalities. ICT intervention helps to reduce the digital divide, which is a challenge to be overcome both domestically (in a given country or region) and internationally (to address gaps between regions, countries, or continents) [15].

In spite of many reports on ICT interventions [12,16–18] in several segments of society (e.g., among elders, students, teachers, young entrepreneurs, civil servants, and residents of rural and isolated areas), without proper investigation of the impact of these initiatives, it is difficult to determine whether the intervention has been successful. Thus, monitoring and evaluating ICT interventions, and even measuring the digital divide, are fundamental for helping managers, researchers, and professionals to make decisions for overcoming the challenges often posed by endemic problems in a region or country. There are many indications that, in developing countries, these endemic problems hinder both the completion of technological innovations and the realization of expected benefits [1,19].

In the literature, we can find successful investigations that evaluate the impact of ICT interventions at the micro-level—that is, they were performed with references to the beneficiaries of the actions in their local context [1,12,16,18,20–22]. These investigations [12,16,20,22] were all carried out by means of questionnaires and/or interviews and were applied to a small number of respondents, thus favoring a detailed view of the impact of ICT interventions within a particular social context. Such an approach, which is costly in terms of physical and human resources, and which would require a long time to complete, is inappropriate for interventions in an extensive geographical territory. However, large-scale assessments are often standardized and do not consider the different conditions of access or the socioeconomic and cultural levels of the participants. In short, a gap exists in the strategies, models, or frameworks necessary for evaluating the impact and monitoring of large-scale ICT interventions. Our research aims to fill the gap in the monitoring of large-scale training programs for digital inclusion.

The objective of the present study is to monitor and identify problems in a large-scale training project throughout its execution. An approach based on Social Network Analysis (SNA) is applied to the data from the training at Telecentros.BR [23], which certifies individuals in using ICT resources. Our analysis relates the influence of the social network of participants to the success of the training, considering local indicators of the telecommunications infrastructure and the socio-economic conditions.

## Setting and Methods

The Brazilian government funded the Telecentros.BR Program [23] at the national level as part of a public policy for digital inclusion in Brazil in different regions of the country (North, Northeast, South, Southeast, and Central-West). This program is focused on installing telecenters and training individuals to disseminate and use ICT in these areas. Community members who can assume the role of digital inclusion agents use the telecenters, assisting others in learning to use ICT resources as a means to improve the social conditions of their communities. For this, the Telecentros.BR provides training that uses a learning platform along with online social networks. Tutors train community members to act as digital inclusion agents in a model originally designed to qualify approximately 16,000 individuals. This program is ideal for our case study because of its large scale in relation to the geographic scope and number of participants involved, with each region having a training facility, known as a “center,” which is responsible for training in its region.

The Telecentros.BR training is structured in phases, with the goals for each phase as follows: in phase 1, to become familiar with the learning platform; in phase 2, to understand and discuss relevant issues (e.g. basic computing, digital inclusion in communities, communication and sharing in networks, digital culture, e-waste, open and free software); and in phase 3, to formulate, carry out, and achieve visibility for projects involving the communities around the telecenters.

To monitor this intervention, the following stakeholders were identified: community members, who are the beneficiaries of the intervention; and, tutors, the agents who diffuse the use of ICT resources among community members (e.g., use of e-gov services, digital content, online social networks). ICT interventions occur in locales where community members live (e.g. neighborhood, city, state, country).

### Data sources

We used the Telecentros.BR dataset to collect information on the participants’ training. This dataset was obtained directly from the implementing organization as an anonymized set of records over an eleven-month period between 2011 and 2012, and full permission was granted by the Federal University of Pará (*Universidade Federal do Pará*) for its use in our analysis. To ensure privacy, participants’ individual data were anonymized by the implementing organization. In the anonymized dataset, a hashed ID identifies each participant. The dataset contains information with respect to the following: (i) exchange of instant messages in the learning platform; (ii) attributes of participants; (iii) participant evaluations; (iv) telecenter where the community member acts.

The dataset contains information about the exchange of instant messages in the learning platform but does not contain the content of the text messages. Thus, each registry represents an online interaction in the following format: <*id_sender*, *id_receiver>*. Looking at both the *id_sender* and *id_receiver* allows us to study the relationships in the social networks constructed by placing edges between nodes that represent participants whenever two participants exchange messages. The social network has 4,382 nodes and 104,831 edges.

The Telecentros.BR dataset also contains certain attributes of the participants as described in Table 1.

**Table 1:** Categories of Attributes of the Telecentros.BR Dataset.

Categories of attributes	Description
Participant’s function	Represented by the attribute *role*, which identifies the participant’s role in the learning platform and can assume one of the following values: tutor, community member, or not identified.
Regional training center	Represented by the attribute *center*; it identifies the center (and region) where the participant performs the training. It can assume one of these values: North, South, Central-West, Northeast, Ceará, Southeast, and São Paulo. Training managers belong to the Coordination center–not represent any specific region of the country.
Locale	Represented by the attribute *municipality*, which identifies the municipality where the participant lives.
Certification	Represented by the attribute *certified*. It identifies whether the participant has successfully concluded the training and can assume the values “yes” or “no.”

Additionally, we used anonymized information from the evaluation system of the Telecentros.BR program. This system contains descriptions from the tutor with respect the performance of a community member at different phases of training.

Because of the lack of data sources for indicators by district, we considered the municipalities as the “locale.” Thus, for each municipality in the Telecentros.BR dataset, we collected education and income indicators from the Atlas of Human Development in Brazil (*Atlas do Desenvolvimento Humano no Brasil*; http://www.atlasbrasil.org.br). These indicators are components of the Municipal Human Development Index (MHDI) and are classified by the Institute for Applied Economic Research (*Instituto de Pesquisa Econômica Aplicada*) [24]. In addition, we obtained information about low-cost Internet access with a speed of at least 1 megabit per second in Brazilian municipalities from the Brazilian Communication Ministry (*Ministério das Comunicações do Brasil;* http://www.mc.gov.br/DSCOM/view/Principal.php). The information on households that have computers with Internet access in Brazilian municipalities was collected from the Demographic Census of the Brazilian Institute of Geography and Statistics (*Instituto Brasileiro de Geografia e Estatística—IBGE*; http://loja.ibge.gov.br/populacao/universo.html). Table 2 presents the categories and ranges of values for these indicators.

**Table 2:** Indicators for Brazilian Municipalities.

Indicator	Description	Category	Values
MHDI income	Represented by the attribute *mhdi_income* and corresponds to the component “income” of the MHDI. It is measured by the municipal income per capita—that is, the average income of residents of a given municipality.	Very low	0.000 ≤ MHDI income ≤ 0.499
		Low	0.500 ≤ MHDI income ≤ 0.599
		Medium	0.600 ≤ MHDI income ≤ 0.699
		High	0.700 ≤ MHDI income ≤ 0.799
		Very high	0.800 ≤ MHDI income ≤ 1.000
MHDI education	Represented by the attribute *mhdi_education* and corresponds to the component “education” of the MHDI. This is based on the geometrical average of the frequency sub-index of children and youngsters in school (weight 2/3) and the education sub-index of the adult population (weight 1/3).	Very low	0.000 ≤ MHDI education ≤ 0.499
		Low	0.500 ≤ MHDI education ≤ 0.599
		Medium	0.600 ≤ MHDI education ≤ 0.699
		High	0.700 ≤ MHDI education ≤ 0.799
		Very high	0.800 ≤ MHDI education ≤ 1.000
Popular Internet infrastructure	Represented by the attribute *national_broadband_plan* with respect to satisfying the goal of the government for popular broadband, providing Internet access for a low cost and speed of at least 1 megabits per second.	Yes	"Yes", if the municipality is attended
		No	"No", if the municipality is not attended.
Households with a computer with Internet access	Represented by the attribute *households_internet*, this value is the percentage of households that have a computer with Internet access in the municipality. This value is determined by X / Y * 100, where X is the number of households in the municipality that answered “Yes” to the 2010 census question on “owning a personal computer and Internet access,” and Y is the total number of households in the municipality.	Very low	0.00 ≤ *households_internet* < 7.00
		Low	7.00 ≤ *households_internet* < 12.96
		Medium	12.96 ≤ *households_internet* < 31.58
		High	31.58 ≤ *households_internet* < 58.48

We also used the georeferenced cartographic database of the Brazilian municipalities that is freely available online in SHP format (shapefile) in the Brazilian Institute of Geography and Statistics (http://downloads.ibge.gov.br/downloads_geociencias.htm.).

### Designing the analysis

**Fig 1:**
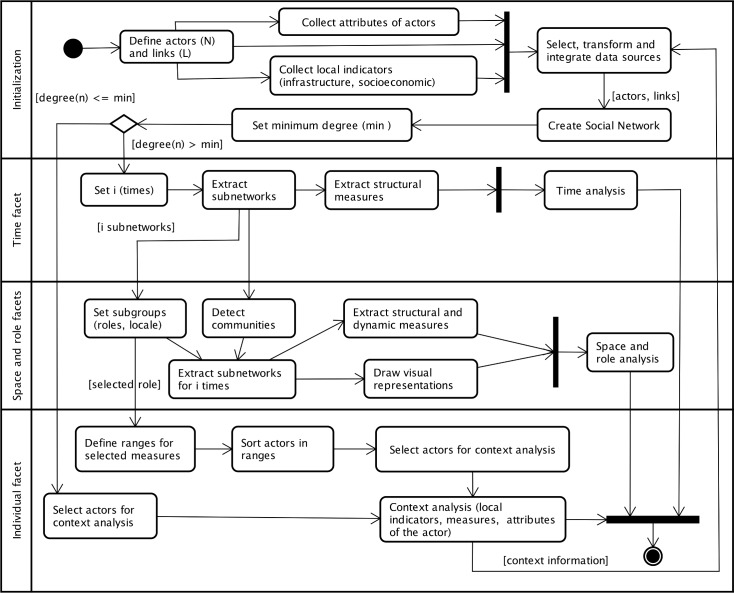
SNA-based Approach for Monitoring ICT Intervention.

Our approach applies the techniques of social network analysis to monitoring and analyzing the outcomes of ICT intervention. The links between individuals are the foundation of our approach because these individuals are in the initial stage of technological appropriation. Thus, we applied our approach to the records of online interaction between participants of the Telecentros.BR training (i) to the time facet, to obtain indicators of the evolution of participation during the training in different moments; (ii) to the space and role facets, to analyze the interactions according to geographic region and the roles of the participants; and (iii) to the individual facet, to favor the identification of actors engaged in training or the identification of variables associated with low participation. Fig 1 shows an overview of our SNA-based approach.

At initialization, the data were prepared and integrated, including the social network of the participants. At this point, we attempted to identify those actors whose number of relationships was not sufficient for a meaningful analysis. In fact, a significant number of community members did not complete the first phase of the training, which was to become familiar with the learning platform. For this, we used the *degree centrality* metric (or simply *degree*) as a measure network activity for a node. *Degree* is defined as the number of links incident upon a node—that is, the number of ties that a node has [25]. We proposed setting a minimum *degree* by which the network under analysis could be reduced so that the actors discarded in this step might be directed to the microanalysis in the step “*select actors for context analysis*.”

For the time facet, we defined the moments of observation (times) according to the three different phases of the training. Thus, one subnetwork was extracted for each time period, and the analysis was performed on the evolution of the structural measurements of *degree centrality* [26] and *density*, expressed as a proportion of the maximum possible number of lines [27–28]. The purpose was to verify whether the time training lends to reducing or to intensifying the interactions between participants.

Because the *role* of the participant in training can influence the social network, we used the *role* attribute to define subgroups for identifying and measuring the interactions between tutors and community members. This classification or clustering of the vertices in the network is done so that each vertex is assigned to exactly one cluster [28], and subnetworks for each time period can be extracted from them. This approach allows extraction of structural measurements only within a particular role for comparison with measurements extracted from another role. In addition to the *density* and *degree* centrality, we used the *closeness* centrality [28], which focuses on the closeness of the participant to other participants. The *closeness centrality* of a vertex is based on the total distance between one vertex and all other vertices, whereby larger distances yield lower closeness centrality scores [28]. The closer a vertex is to all other vertices, the more easily information may reach it and the higher its centrality. In addition to structural measurements, we used the *Watts-Strogatz (WS) clustering coefficient* [29] to quantify the connectivity of actors, considering the tutor and community member subgroups.

Subgroups based on the attribute *center* were also defined for supporting visual representations of the networks of tutors and community members. Visualization of the networks according to centers contributes to identifying actors with more or fewer links to other regions.

One of the goals of the Telecentros.BR training is that as community members appropriate ICT resources, they will be able to build social networks by sharing day-to-day problems and solutions in the telecenters. In this sense, analyses of the centers are used to understand whether interactions between community members are constructed independent of geographical regions. For this purpose, we used a community detection algorithm based on a modularity quality function known as the Louvain method [30–31], which is a heuristic algorithm based on modularity optimization [32].

Community detection is well studied in the literature; many different community detection algorithms have been presented in social network analysis literature, and a good survey of these algorithms can be found in Fortunato [33]. In our approach, we compared the automatically detected communities with the clusters pre-assigned by *center*. We used two types of statistical indices to analyze the association between clusters found by the Louvain method and the clusters pre-assigned by *center*: Cramer’s V and Rajski’s information index [28]. We also used visual representations and dynamic measurements to quantify the connectivity of actors in clusters. Reduction methods were used to understand the network structure and to identify the roles of actors within the communities found.

In view of the context analysis, ranges of values for the extracted measurements were defined, and actors were classified according to these ranges. Community members and *centers* are placed within the top range of *degree* centrality as well as being assigned to the simplest measure of prestige, which is the *degree input*, also known as a measure of popularity [34]—that is, a count of the number of ties directed to the node. This strategy can be used to support the selection of new tutors; however, as the *degree* may be influenced by regional characteristics or the center’s performance in using the social networking platform, we performed the same classification process for the actors within each *center*.

For the set of actors with a *degree* equal to zero and for actors who stopped interactions during the course, we tested some qualitative hypotheses, such as the presence of inappropriate telecommunications infrastructure and sustainability in telecenters, lack of personal interest, and weak training from the center in using the learning platform. Given that we had no access to the participants, the anonymized information from the Telecentros.BR evaluation system served to support our tests of some of these hypotheses.

The training was conducted in different municipalities with certain indicators—for example, socio-economic and infrastructural—that could influence variables in participation (*degree*) and success in training (*certified*). Thus, to study the association between these variables, we advanced from the SNA-based approach to applying the Bayesian networks technique, also referred to as causal networks or graphic models of probabilistic dependence. Bayesian networks are models that encode probabilistic relationships among variables that represent a certain domain. These models include both a qualitative and a quantitative structure. The qualitative structure represents dependencies between nodes (variables), while the quantitative structure represents the conditional probabilities of these nodes. The idea is evaluate the nodes in probabilistic terms [35,36] and to provide a compact and easy-to-use representation of the probabilistic information from the data. We used the K2 heuristic search algorithm [37] to find the most probable Bayesian network structure within the search space. This network structure is an effective way to communicate dependencies among the domain variables.

Based on the attributes of municipalities where the interventions occur (*mhdi_income*, *mhdi_education*, *national_broadband_plan*, *households_internet*), degree centrality (*degree*), region (*center*), and certification of community members (*certified*), we created a dataset as an input file for the Bayesian analysis. Once the network was established, the posterior distribution of the parameters was estimated by statistical inference.

To support the discussion, we used Qgis software version 2.6.0-Brighton (http://qgis.org) to show the geographic distribution for the percentage of households that have computers with Internet access in the Brazilian municipalities.

## Results and Discussion

### Initialization

The integration of data sources revealed that the social network contained 4,382 participants for the duration of the training program (11 months). In this sequence, to make the analysis more relevant, we considered only the nodes having a minimum *degree centrality* equal to one (*degree (node*_*min*_ = 1)—pendants were deleted. Thus, the analysis of the reduced network began with 2,303 nodes.

### Time facet

We determined three different time periods: months 1–2 (#1), months 3–6 (#2), and months 7–11 (#3) after the beginning of the training. These time periods are determined by training phases, such that #1 corresponds to phase 1, #2 corresponds to phase 2 and #3 corresponds to phase 3. For these moments, we computed the *density* and *degree centrality* indices (Table 3).

**Table 3:** Indices of the Telecentros.BR Network.

	#1	#2	#3
Nodes	2,303	2,303	2,303
Links	19,025	37,681	48,125
Density	0.0036	0.0071	0.0091
Degree centrality (average)*	16.5219	32.7234	41.7933

* Value not standardized

In structural terms, the network in #2 is 98% denser than the network in #1, and the network in #3 is 153% denser than the network at the beginning of the training (#1). The increases in density and centrality can be explained by the promotion of physical meetings that stimulated spontaneous interactions through instant messages. This was a desirable development in terms of training progress, given the structure and objectives of each phase of the course.

### Space and role facets

Subgroups were defined to analyze the interactions according to the roles played by the participants (i.e., tutor or community member) or their center, which also indicated the geographical regions of the country. For role subgroups, we generated structural and dynamic indices for the three different moments (Table 4).

**Table 4:** Indices of Networks of Tutors and Community Members.

	Tutors			Community members			Tutors and community members		
	#1	#2	#3	#1	#2	#3	#1	#2	#3
Nodes	210	210	210	2,085	2,085	2,085	2,303	2,303	2,303
Links	2,616	1,077	2,093	3,917	6,020	8,504	19,025	37,681	48,125
Density (no loops)	0.0596	0.0245	0.0477	0.0009	0.0014	0.002	0.0036	0.0071	0.0091
Degree average*	24.9	10.3	19.9	3.8	5.8	8.2	16.5	32.7	41.8
Degree (highest)*	609	118	447	275	394	419	1275	1963	2735
Closeness centrality (average)	0.1713	0.0661	0.0923	0.0109	0.0257	0.0496	0.0416	0.0787	0.1736
WS clustering coefficient	0.4724	0.3571	0.4724	0.0877	0.087	0.0599	0.3192	0.2618	0.2719

* Value not standardized

The creation of clusters for individuals according to their role allowed us to analyze the interactions between peers, excluding interactions in the hierarchical relationship between tutors and community members. During the training process, community members developed projects involving communities around the telecenters. Interactions among participants were encouraged to promote development, with the aim being to map existing problems and to articulate solutions that would enhance the use of resources and increase community participation.

In structural terms, in the three analyzed time periods, the network of community members proved to be less dense than the networks of both the tutors and all the participants. The high density of the network of tutors is likely because they met physically more often than did community members, who were involved in the online training most of the time. Nevertheless, the network of community members increased more in #3 in relation to #1 when compared to the network of tutors during the same time period. This result was expected because phase 3 was intended to encourage interactions in order to give visibility to the project developed by the participants.

The *closeness centrality* (*average*), which focuses on the closeness of a participant in relation to other participants, is lower in the network of community members than in the network of tutors; this result was expected because many of the tutors were acquainted with each other, unlike the community members. However, when we considered the evolution of this index between #1 and #3, we verified that it increased in the network of community members while decreasing in the network of tutors. This was a desirable result because the training was intended to bring the community members closer each other to help solve the day-to-day problems of telecenters.

**Fig 2:**
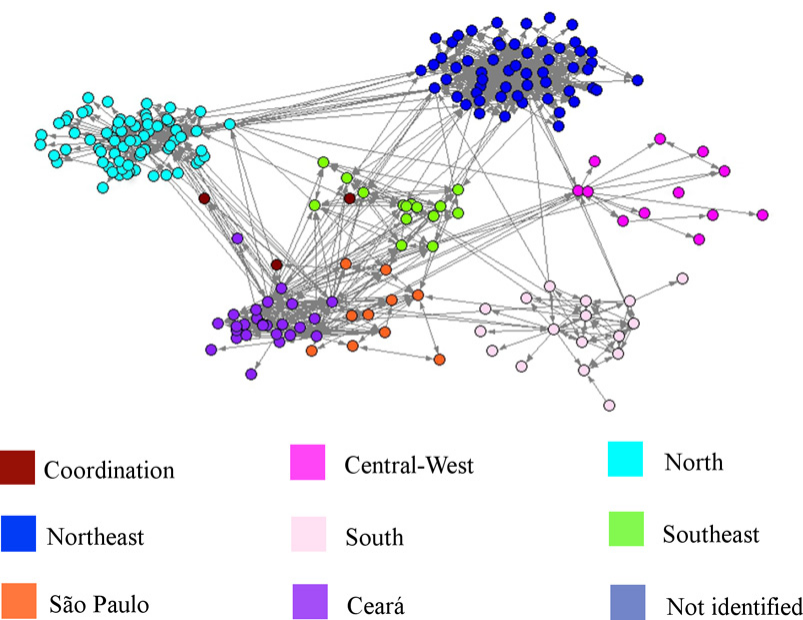
Network of Relationships Among Tutors.

**Fig 3:**
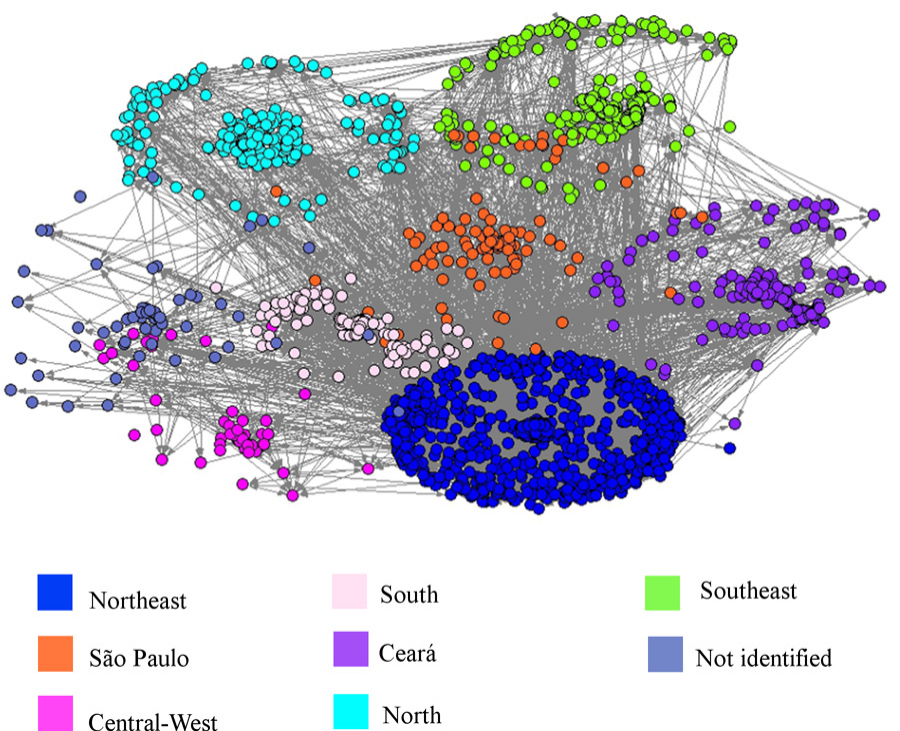
Network of Relationships Among Community Members.

Among the dynamic measures, the *WS clustering coefficient* for the network of tutors is higher than that for community members. These highest values in the network of tutors reflect a high degree of transitivity that can be explained by the existing relationships among tutors of same center. The groupings of tutors and community members in the same region are visualized in Figs 2 and 3, respectively. The individuals are grouped by centers (clusters), which are identified by different colors.

Analyses employing the *center* are used to understand whether the interactions between community members are constructed independently of geographical regions. For this, we used a community detection algorithm based on a modularity quality function known as the Louvain method.

From the visualization, we used an automatic community detection method to identify whether the interactions between participants were constructed independently of geographical regions. For the network with all participants during the 11 months of training, we applied the Louvain method with a resolution parameter equal to 1, finding 30 clusters (ID-1 to ID-30) with modularity equal to 0.8094. Using the Cramer’s V statistical index to analyze the association between the output of the Louvain method (30 clusters) and the clusters pre-assigned by *center* (9 clusters), we obtained the value 0.7375, which indicates strong association. However, because the cross-tabulation contains many cells that are (nearly) empty, this index is not very reliable. Thus, we used the Rajski’s information index to measure the degree of association between the two classifications. The strongest correlation (0.7413) of the Rajski’s index indicated that the classification of the cluster by *center* could be predicted by classifying with the Louvain method. For example: in cluster ID-1, found by the Louvain method, 96.8% of the nodes are from the North and in cluster ID-3, 72% are from the Northeast. Table 5 presents the percentage of nodes from the clusters pre-assigned by center (9 clusters) that coincides with the output of the Louvain method (30 clusters).

**Table 5:** Cross-tabulation (%) between the clusters (Louvain method and pre-assigned by center).

	Cluster ID (pre-assigned by center)^*^									
Cluster ID (Louvain method)	1	2	3	4	5	6	7	8	9	*WS clustering coefficient*
1	0.8	0.8	96.8	0.0	0.0	1.6	0.0	0.0	0.0	0.5743
2	1.0	1.0	0.0	1.0	0.0	1.5	1.5	93.1	1.0	0.7453
3	0.0	0.7	5.8	72.5	1.4	4.3	0.5	14.7	0.0	0.2335
4	0.0	0.7	0.7	2.9	94.2	0.7	0.0	0.7	0.0	0.5900
5	0.0	0.4	0.0	1.2	0.0	98.0	0.0	0.4	0.0	0.3063
6	0.0	68.1	1.4	0.0	1.4	5.6	0.0	23.6	0.0	0.5146
7	0.0	0.0	96.3	1.9	0.6	1.2	0.0	0.0	0.0	0.5898
8	0.0	0.0	4.3	91.3	0.0	2.2	0.0	2.2	0.0	0.6817
9	0.0	0.0	3.1	84.4	0.0	0.0	3.1	9.4	0.0	0.5286
10	0.0	0.7	0.0	1.4	10.7	1.4	83.6	2.1	0.0	0.6996
11	0.0	0.0	0.0	91.2	0.0	5.9	0.0	2.9	0.0	0.6395
12	0.0	0.0	2.8	97.2	0.0	0.0	0.0	0.0	0.0	0.3787
13	0.0	2.7	5.4	78.4	0.0	0.0	0.0	13.5	0.0	0.6691
14	0.0	0.0	3.3	93.3	0.0	0.0	0.0	3.3	0.0	0.6755
15	0.0	0.0	0.0	93.8	1.6	1.6	1.6	1.6	0.0	0.4837
16	0.0	0.0	0.0	97.1	0.0	0.0	2.9	0.0	0.0	0.7714
17	0.0	0.0	0.0	100.0	0.0	0.0	0.0	0.0	0.0	0.8636
18	0.0	0.0	0.0	94.1	0.0	5.9	0.0	0.0	0.0	0.6902
19	0.0	0.0	0.0	96.9	0.0	3.1	0.0	0.0	0.0	0.7323
20	0.0	3.2	0.0	96.8	0.0	0.0	0.0	0.0	0.0	0.7076
21	0.0	0.0	2.4	95.2	0.0	2.4	0.0	0.0	0.0	0.6321
22	0.0	0.0	1.7	86.4	5.1	6.8	0.0	0.0	0.0	0.4213
23	0.0	0.0	0.0	93.5	3.2	3.2	0.0	0.0	0.0	0.6683
24	0.0	1.8	0.0	98.2	0.0	0.0	0.0	0.0	0.0	0.6372
25	0.0	0.0	0.0	96.6	0.0	3.4	0.0	0.0	0.0	0.4511
26	0.0	0.0	0.0	88.6	0.0	5.7	0.0	5.7	0.0	0.7160
27	0.0	0.0	0.0	100.0	0.0	0.0	0.0	0.0	0.0	0.8524
28	0.0	0.0	0.0	100.0	0.0	0.0	0.0	0.0	0.0	0.8441
29	0.0	0.0	0.0	50.0	0.0	50.0	0.0	0.0	0.0	-
30	0.0	0.0	0.0	0.0	0.0	100.0	0.0	0.0	0.0	-

^*^ (1) Coordination; (2) Centre-west; (3) North; (4) Northeast; (5) South; (6) Southeast; (7) São Paulo; (8) Septentrional Northeast; (9) Not identified.

The largest cluster (ID-3), with 414 nodes, presented the lowest *WS clustering coefficient* (0.2335). Among the 14 clusters with a *WS clustering coefficient* above the average (0.6178), we found 12 clusters with a predominance of members from the Northeast, one with a predominance of members from Ceará, and one with a predominance of members from São Paulo.

**Fig 4:**
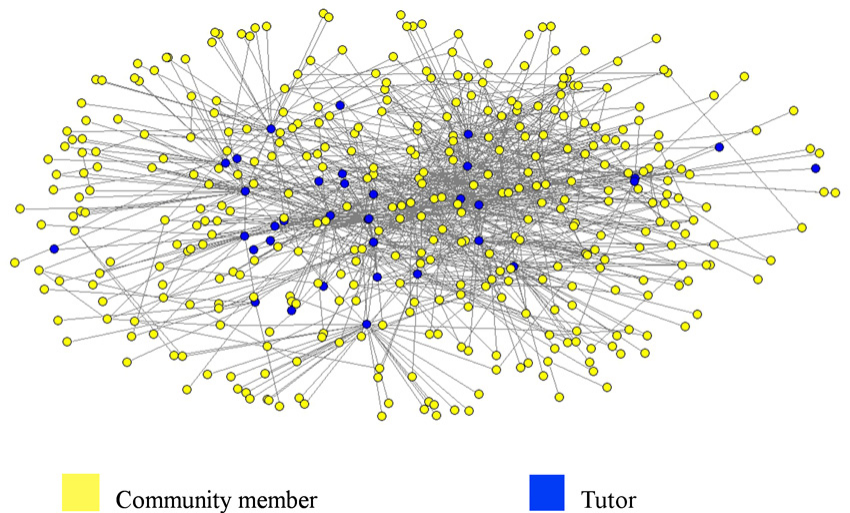
Network of Cluster ID-3 (Louvain method).

**Fig 5:**
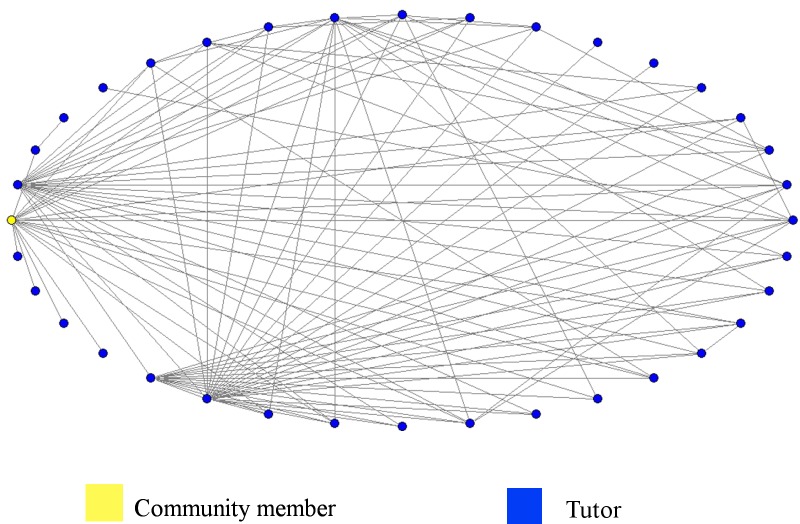
Network of Cluster ID-3 (Louvain method) After the Shrinking Operation.

We next used some reduction methods to understand the network structure and to identify the roles of actors within the clusters found by the Louvain method. The subnetwork extraction for each cluster offers a local view that includes the roles and centers of actors. Using this local view, we shrank the community members into one new vertex favoring the analysis of the relation between tutors and community members. As an example, Fig 4 shows cluster 3 with 414 nodes (35 tutors and 379 community members) and Fig 5 shows the same cluster after the shrinking operation. The color of the vertex determines the role of the actor in these figures. We also observe that tutors are present in the networks extracted from cluster ID-1 to ID-28.

### Individual facet

Considering the beneficiaries of the action (selected role = “community members”) with the highest level of participation, we focused on actors with the highest value of *degree centrality* and *degree prestige*. Table 6 presents the ranges of *degree centrality* and *degree prestige*, respectively. These ranges were defined according to the frequency of states of the values of these indices. The states of the variables represent the possible values that a variable can assume.

**Table 6:** Ranges of Values of *Degree Centrality* and *Degree Prestige*.

Range of *degree centrality*	Range of *degree prestige*	Community members
[0.0002 to 0.0015)	[0.0000 to 0.0022)	25%
[0.0015 to 0.0063)	[0.0022 to 0.0091)	25%
[0.0063 to 0.0167)	[0.0091 to 0.0243)	25%
[0.0167 to 0.1681]	[0.0243 to 0.2094]	25%

Thus, to analyze the participants with the highest *degree centrality*, we considered only the top range (25%) with 524 actors. The same process was applied to *degree prestige*—that is, considering the 526 actors in the top range (25%). When both lists were combined and the repeated entries removed, the final list included 572 actors. This experiment was very useful for identifying actors who would be able to act as tutors in new training courses. It also served as a strategy to clarify the importance of interactions in large-scale digital inclusion training programs to parties who invest in ICT interventions based on telecenters.

Among these 572 participants, we found 416 community members from the Northeast but a low number of members from the other regions. This result may be influenced by regional characteristics or by different training strategies in the social networking platform. Thus, we considered the ranges of values for each *center*. This strategy promotes analysis by training center, aiming to minimize the influence of inequality between regions, because it delimits the scope of the geographic area in question. Indeed, for the North we found other ranges of values (Table 7) and 71 actors in the top range of *degree centrality* or *degree prestige*. The strategy of using clusters to identify actors in the regions was shown to be relevant because it allows us to reduce the scope of the analysis. We think that this strategy may lend itself to even greater refinement of the analysis if we add clusters by municipalities or districts.

**Table 7:** Ranges of Values of *Degree Centrality* and *Degree Prestige* (North).

Ranges of *Degree Centrality*	Range of *Degree Prestige*	Community members (North)
[0.0002 to 0.0017)	[0.0000 to 0.0026)	25%
[0.0017 to 0.0059)	[0.0026 to 0.0091)	25%
[0.0059 to 0.0117)	[0.0091 to 0.0182)	25%
[0.0117 to 0.1027]	[0.0182 to 0.1134]	25%

As described in the initialization section, the number of actors without any interaction was significant. Additionally, we identified 152 nodes that did not create new interactions from time #1 to time #2 and 256 nodes from time #2 to time #3. For these cases, we offer some hypothetical reasons for lack of interaction: inappropriate telecommunications infrastructure in telecenter; problems of sustainability, such as telecenters that cannot maintain their infrastructure (energy, water, and maintenance); weak training provided by the center in the learning platform; low sociocultural identification with the program; lack of interest in the community or from the managers, resulting in partial or total closure of the telecenter; lack of personal interest; difficulty in delivery and replacement of equipment, especially in isolated communities in rural areas or banks of rivers, such as Amazonian peoples.

To test some of these hypotheses, we resorted to the analyses of participant performance as conducted by tutors. We found reports about community members who, although registered in the learning platform, did not initiate training because the telecenter did not receive the equipment or because, though it received equipment, the latter was not operational. Such was the case for the telecenter in the town of Pacajá (population 41,000 and 600 km away from Belém, the capital of the State of Pará, North of Brazil). It took months to deliver the machines because of weather conditions, problems with transport, and theft of machines by pirates (i.e., Amazon River thieves). In fact, owing to these challenges, the original goal of installing telecenters was not achieved in several municipalities, as revealed in a report elaborated by individuals of the civil society: from a total of 8,083 expected telecenters, only 1,193 came into operation, and 2,800 received the equipment without its being installed [38].

We also found 206 reports about low participation of community members because of unstable connections or low Internet speed in 96 telecenters in the North; 40, Northeast; 34, Ceará; 13, Central-West; 10, São Paulo; 8, South; and 5, Southeast. As one example, the telecenter in the indigenous Zoró, which is located in the countryside of the state of Rondônia in northwestern Brazil, does not have Internet connections. Therefore, to access the online learning platform, the community member went to a neighboring town to use an Internet cafe.

The tutors’ analyses of participants’ performance also showed a significant number of reports (149) that revealed a lack of interest on the part of the participants, with 72 in the North; because we had no access to the participants, we could not investigate the reasons for this. However, as the training was performed in different municipalities, the variables of infrastructure and socioeconomic characteristics may have been influential, which is treated in the analysis of scenarios presented in next section.

### Analysis of scenarios

**Fig 6:**
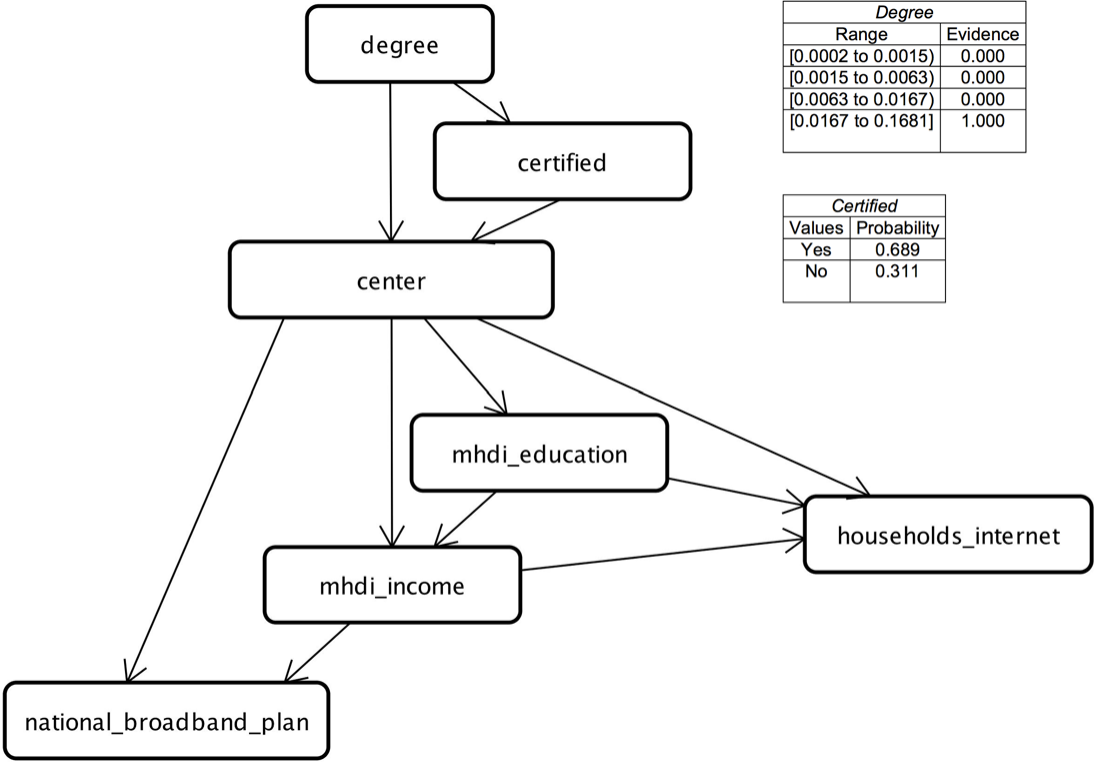
Structure of the Bayesian Network.

To set up the learning of the Bayesian network structure, we use the dataset composed of the variables *mhdi_education*, *mhdi_income*, *center*, *households_internet*, *degree*, *national_broadband_plan*, and *certified*. Fig 6 presents the resultant Bayesian network for this dataset according to the values and ranges of values defined in Tables 1, 2 and 5.

The associations allowed us to measure, in probabilistic values, the influence of the *degree* of the participant on finishing the training: given evidence that the *degree* of a community member is in the highest range (over 0.0166), the probability that this individual will finish the training is 0.689 (Fig 6). However, this probability falls to 0.166 when there is evidence that the *degree* is in the lower range (*degree* < 0.0015).

When we consider the socio-economic (*mhdi_education and mhdi_income*) and infrastructural (*households_internet and national_broadband_plan*) indicators of municipalities, we observe that in the North and Northeast regions, the most frequent scenario is for municipalities not to have the National Broadband Plan, these regions having the lowest percentage of households with a computer that has Internet access, as well as the lowest MHDI income and MHDI education. For example, given the evidence that the region is the Northeast, the probability that one municipality in this region will have a high percentage of households that have a computer with Internet access is 0.099 (*households_Internet* > 31.57%); the probability that *national_broadband_plan* = *no* is 0.871; the probability that *mhdi_education* = low or *mhdi_education* = very low is 0.805; and the probability that *mhdi_income* ≤ 0.699 (low, very low, medium) is 0.872. However, when the evidence indicates that the municipality belongs to the North region, the probability of the highest percentage of households’ having a computer with Internet access is 0.0; the probability that *national_broadband_plan* = *no* increases to 0.917; the probability that *mhdi_education* = low or *mhdi_education* = very low is 0.574; and the probability that *mhdi_income* ≤ 0.699 (low, very low, medium) is 0.715.

In the highest range of *degree*, an individual belonging to the Northeast (0.739) has a greater probability of belonging than if the participant is in the North region (0.067). In addition, the Northeast has a higher probability that *certified = yes* (0.419) than does the North (0.273). We think this result is evidence that in the Northeast center, the social networking platform is most widely used, contributing to the probability that a greater number of individuals will be certified in training.

When the evidence is that a community member belongs to the Southeast region, the probability of completion of the training is 0.609, higher than in all other regions. However, this probability falls to 0.441 when there is evidence that the *degree* is in the lower range (*degree* < 0.0015). For municipalities in the Southeast, the probabilities are as follows: 0.615 for the highest percentage of households with computer with Internet access; 0.626 for *national_broadband_plan* = *yes*; 0.732 if the MHDI income is high or very high (*mhdi_income* ≥ 0.700); and 0.590 if the MHDI education is high.

**Fig 7:**
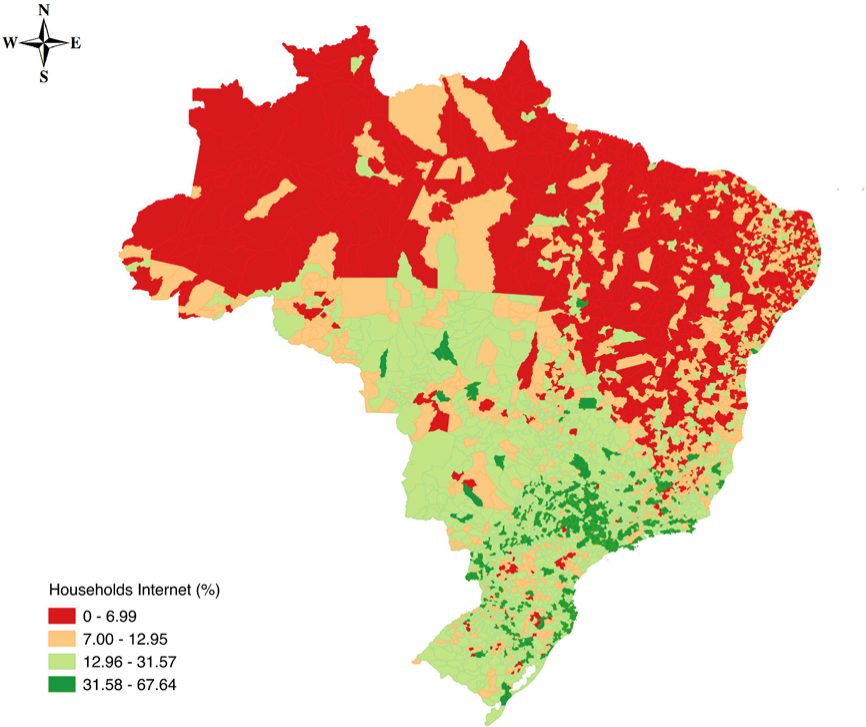
Percentage of Households with Computer with Internet Access in Brazilian Municipalities.

The North and Northeast regions are those having the most municipalities without access to the National Broadband Plan and where the percentage of households that have a computer with Internet access is lower than 7% (Fig 7). However, according to our study, the Northeast is also the center with the highest probability that a community member has a higher *degree*.

## Conclusions

In this paper, we applied an SNA-based approach to monitor and identify problems in ICT intervention. The approach we presented, based on social network analysis and mining, is applicable to training that will target and certify a large number of participants in different geographical regions. Our SNA-based approach proved to be complementary to approaches that consider only individual access to technology, as the two approaches together take into account both the perspective of the beneficiaries and that of the experts and funders of the program.

Our study reveals that a significant number of participants had low or no interaction; likewise, the tutors reported weak infrastructure in terms of implementing training for 206 of the community members. For these community members, we observed that their municipalities are small populations, often located in the north, northeast, and center-west of the country. Although these cases represent a very small part of the target audience of the intervention, they reveal scenarios wherein online training is still unthinkable until infrastructure challenges can be overcome. Our results show that increasing the success of ICT intervention in these locales depends fundamentally on reducing the inequalities in the country. These results corroborate the arguments from the literature [1,39].

Despite the high concentration of digitally excluded individuals in the North regions of the country, we observed that this region presented the lowest probability of certified participants in the Telecentros.BR training. By way of contrast, the Northeast, which served municipalities with similar characteristics, presented a high probability of certification, associated with the highest degree in the social networking platform.

For developing countries such as Brazil, where almost half of the population resides in urban and rural areas, and has never accessed the Internet before, there remains interest in large-scale ICT projects to combat the digital divide, but even these initiatives are criticized. Moreover, with the expanding use of social networks, SNA-based approaches to monitoring ICT interventions seem to be promising. However, there are many challenges in adopting an approach for large-scale intervention because many aspects must be considered. It is not always possible, for example, to perform analyses for a better understanding of social phenomena in their context because of lack of data or human resources. More studies are needed for better comprehension of the influence of these interventions in the involved communities. This comprehension can assist in the decision to maintain investments in localities where the impact of innovation and its consequent social transformation in the communities is significant.

Finally, our study has demonstrated that large-scale ICT interventions may provide promising scenarios for the study of failure in developing countries [1,19,40].

## Supporting Information

S1 File Indicators for Brazilian Municipalities. (XLSX)

## References

[1] Avgerou C. Information systems in developing countries: a critical research review. J Inf Technol. 2008;23:133–46. 10.1057/palgrave.jit.2000136

[2] Farhadi M, Ismail R, Fooladi M. Information and communication technology use and economic growth. PLoS ONE. 2012;7:e48903 10.1371/journal.pone.0048903 23152817 PMC3495961

[3] Datta P. A preliminary study of ecommerce adoption in developing countries. Inform Syst J. 2011;21:3–32. 10.1111/j.1365-2575.2009.00344.x

[4] White GRT, Afolayan A, Plant E. Challenges to the adoption of e-commerce technology for supply chain management in a developing economy: a focus on Nigerian SMEs In: Lacka E, Chan HK, Yip N, editors. E-commerce platform acceptance: suppliers, retailers and consumers. Switzerland: Springer International Publishing; 2014 pp. 23–39.

[5] Basu S. E-government and developing countries: an overview. Int’l Rev L Comp & Tech. 2004;18:109–32. 10.1080/13600860410001674779

[6] Furuholt B, Matotay E. Supply and demand of e-government services in developing countries: cases from Tanzania In: Pooley R, Coady J, Schneider C, Linger H, Barry C, Lang M, editors. Information systems development: reflections, challenges, and new directions. New York: Springer New York; 2013 pp. 63–76.

[7] Andrade AD, Urquhart C. Unveiling the modernity bias: a critical examination of the politics of ICT4D. Inf Technol Dev. 2012;18:281–92. 10.1080/02681102.2011.643204

[8] Asgarkhani M. Digital government and its effectiveness in public management reform. Public Manag Rev. 2005;7:465–87. 10.1080/14719030500181227

[9] Heeks R, Bailur S. Analyzing e-government research: perspectives, philosophies, theories, methods, and practice. Gov Inform Q. 2007;24:243–65. 10.1016/j.giq.2006.06.005

[10] McLean S, Sheikh A, Cresswell K, Nurmatov U, Mukherjee M, Hemmi A, et al The impact of telehealthcare on the quality and safety of care: a systematic overview. PLoS ONE. 2013;8: e71238 10.1371/journal.pone.0071238 23977001 PMC3747134

[11] Wong AMK, Chang W-H, Ke P-C, Huang C-K, Tsai T-H, Chang H-T, et al Technology acceptance for an intelligent comprehensive interactive care (ICIC) system for care of the elderly: a survey-questionnaire study. PLoS ONE. 2012;7:e40591 10.1371/journal.pone.0040591 22870200 PMC3411612

[12] Ashraf M, Hanisch J, Swatman P. ICT intervention in the ‘Chandanbari’ Village of Bangladesh: results from a field study. Inform Syst Front. 2009;11:155–66. 10.1007/s10796-008-9133-0

[13] Undp. Human Development Report 2001: Making new technologies work for human development 2001 New York: United Nations Development Programme Available: http://hdr.undp.org/sites/default/files/reports/262/hdr_2001_en.pdf.

[14] Luna-Reyes LF, Gil-Garcia JR, Romero G. Towards a multidimensional model for evaluating electronic government: Proposing a more comprehensive and integrative perspective. Gov Inform Q. 2012;29:324–34. 10.1177/0193841X0002400404

[15] Mutula SM, Brakel van P. An evaluation of e-readiness assessment tools with respect to information access: towards an integrated information rich tool. Int J Inform Manage. 2006;26:212–23. 10.1016/j.ijinfomgt.2006.02.004

[16] Teles A, Joia LA. Assessment of digital inclusion via the actor-network theory: the case of the Brazilian municipality of Piraí. Telemat Inform. 2011;28:191–203. 10.1016/j.tele.2010.09.003

[17] Calvani A, Fini A, Ranieri M, Picci P. Are young generations in secondary school digitally competent? A study on Italian teenagers. Comput Educ. 2012;58:797–807. 10.1016/j.compedu.2011.10.004

[18] Venkatesh V, Sykes TA. Digital divide initiative success in developing countries: A longitudinal field study in a village in India. Inform Syst Res. 2013;24:239–60. 10.1287/isre.1110.0409

[19] Pscheidt M. Appropriate information system development: A methodology for sustainable cross-cultural information system production and use. D.Sc. Thesis, The Radboud Universiteit Nijmegen. 2012. Available: http://www.cs.ru.nl/M.Pscheidt/thesis.pdf.

[20] Ashraf M, Swatman P, Hanisch J. An extended framework to investigate ICT impact on development at the micro (community) level. Proceedings of 16^th^ European Conference on Information Systems (ECIS). 2008; Galway, Ireland.

[21] Adam L, Wood F. An investigation of the impact of information and communication technologies in sub-Saharan Africa. J Inf Sci. 1999;25:307–18. 10.1177/016555159902500407

[22] Chang S-I, Yen DC, Chang I-C, Chou J-C. Study of the digital divide evaluation model for government agencies–a Taiwanese local government’s perspective. Inform Syst Front. 2012;14:693–709. 10.1007/s10796-011-9297-x

[23] Brazil. Decree n. 6991, National program to support digital inclusion in the communities—Telecentros.BR. In: Republic Pot, editor. DOU n. 206, Sec.1, p. 3: Brazilian National Press; 2009.Available:http://pesquisa.in.gov.br/imprensa/jsp/visualiza/index.jsp?jornal=1&pagina=3&data=28/10/2009.

[24] IPEA. Índice de Desenvolvimento Humano Municipal Brasileiro. Brasília: Programa das Nações Unidas para o Desenvolvimento—PNUD; 2013 96 p. Portuguese. Available: http://www.ipea.gov.br/portal/images/stories/PDFs/130729_AtlasPNUD_2013.pdf

[25] Wasserman S, Faust K. Social network analysis: Methods and application Cambridge, UK: Cambridge University Press; 1994.

[26] Borgatti SP. Centrality and network flow. Soc Networks. 2005;27:55–71. 10.1016/j.socnet.2004.11.008

[27] Rowley TJ. Moving beyond dyadic ties: a network theory of stakeholder influences. Acad Manage Rev. 1997;22:887–910. 10.5465/AMR.1997.9711022107

[28] De Nooy W, Mrvar A, Batagelj V. Exploratory social network analysis with Pajek 2nd ed. New York: Cambridge University Press; 2005.

[29] Watts DJ, Strogatz SH. Collective dynamics of ‘small-world’ networks. Nature. 1998;393:440–2. 10.1038/30918 9623998

[30] Blondel VD, Guillaume J-L, Lambiotte R, Lefebvre E. (2008). Fast unfolding of communities in large networks. J Stat Mech-Theory E. 2008;10:P10008 10.1088/1742-5468/2008/10/P10008

[31] Rotta R, Noack A. Multilevel local search algorithms for modularity clustering. J Exp Algorithms. 2011;16:article 2.3. 10.1145/1963190.1970376

[32] Newman MEJ, Girvan M. Finding and evaluating community structure in networks. Phys Rev E. 2004;69:026113 10.1103/PhysRevE.69.026113 14995526

[33] Fortunato S. Community detection in graphs. Phys Rep. 2010;486:75–174. 10.1016/j.physrep.2009.11.002

[34] Faust K, Wasserman S. Centrality and prestige: A review and synthesis. J Quant Anthropol. 1992;4(1):23–78.

[35] Chen Z. Data mining and uncertain reasoning: an integrated approach New York: John Wiley & Sons; 2001.

[36] Korb KB, Nicholson AE. Bayesian artificial intelligence 2nd ed. London, UK: Chapman & Hall/CRC Press; 2010.

[37] Cooper GF, Herskovits E. A Bayesian method for the induction of probabilistic networks from data. Mach Learning. 1992;9(4): 309–47. 10.1007/BF00994110

[38] Lopes Á. 11^th^ Digital Inclusion Workshop: Letter to President Dilma Rousseff. ARede. 2012. Available:http://www.revista.arede.inf.br/site/component/content/article/237-acontece/acontece-2012/5203-11-oid-comemora-conquistas-da-sociedade-civi-e-faz-reivindicacoes-a-presidente-dilma-rousseffdilma.

[39] Heeks R, Kenny C. The economics of ICTs and global inequality: convergence or divergence for developing countries? In: Group DI, editor. Development Informatics Working Paper Series. 10a Manchester, UK: Institute for Development Policy and Management; 2002 Available: http://www.seed.manchester.ac.uk/medialibrary/IDPM/working_papers/di/di_wp10a.pdf

[40] Braa J, Monteiro E, Sahay S. Networks of action: sustainable health information systems across developing countries. Manag Informat Syst Q. 2004;28:337–62.

